# Prognostic Prediction Using a Stemness Index-Related Signature in a Cohort of Gastric Cancer

**DOI:** 10.3389/fmolb.2020.570702

**Published:** 2020-09-04

**Authors:** Xiaowei Chen, Dawei Zhang, Fei Jiang, Yan Shen, Xin Li, Xueju Hu, Pingmin Wei, Xiaobing Shen

**Affiliations:** ^1^Key Laboratory of Environmental Medicine Engineering, Ministry of Education, School of Public Health, Southeast University, Nanjing, China; ^2^Department of Epidemiology and Health Statistics, School of Public Health, Southeast University, Nanjing, China; ^3^Nanjing Municipal Center for Disease Control and Prevention, Nanjing, China

**Keywords:** gastric cancer, cancer stem cells, mRNAsi, TCGA, WGCNA, LASSO regression, prognosis

## Abstract

**Background:**

With characteristic self-renewal and multipotent differentiation, cancer stem cells (CSCs) have a crucial influence on the metastasis, relapse and drug resistance of gastric cancer (GC). However, the genes that participates in the stemness of GC stem cells have not been identified.

**Methods:**

The mRNA expression-based stemness index (mRNAsi) was analyzed with differential expressions in GC. The weighted gene co-expression network analysis (WGCNA) was utilized to build a co-expression network targeting differentially expressed genes (DEG) and discover mRNAsi-related modules and genes. We assessed the association between the key genes at both the transcription and protein level. Gene Expression Omnibus (GEO) database was used to validate the expression levels of the key genes. The risk model was established according to the least absolute shrinkage and selection operator (LASSO) Cox regression analysis. Furthermore, we determined the prognostic value of the model by employing Kaplan-Meier (KM) plus multivariate Cox analysis.

**Results:**

GC tissues exhibited a substantially higher mRNAsi relative to the healthy non-tumor tissues. Based on WGCNA, 17 key genes (ARHGAP11A, BUB1, BUB1B, C1orf112, CENPF, KIF14, KIF15, KIF18B, KIF4A, NCAPH, PLK4, RACGAP1, RAD54L, SGO2, TPX2, TTK, and XRCC2) were identified. These key genes were clearly overexpressed in GC and validated in the GEO database. The protein-protein interaction (PPI) network as assessed by STRING indicated that the key genes were tightly connected. After LASSO analysis, a nine-gene risk model (BUB1B, NCAPH, KIF15, RAD54L, KIF18B, KIF4A, TTK, SGO2, C1orf112) was constructed. The overall survival in the high-risk group was relatively poor. The area under curve (AUC) of risk score was higher compared to that of clinicopathological characteristics. According to the multivariate Cox analysis, the nine-gene risk model was a predictor of disease outcomes in GC patients (HR, 7.606; 95% CI, 3.037–19.051; *P* < 0.001). We constructed a prognostic nomogram with well−fitted calibration curve based on risk score and clinical data.

**Conclusion:**

The 17 mRNAsi-related key genes identified in this study could be potential treatment targets in GC treatment, considering that they can inhibit the stemness properties. The nine-gene risk model can be employed to predict the disease outcomes of the patients.

## Introduction

Gastric cancer (GC) is a leading cause of morbidity and death globally. According to the GLOBOCAN 2018 estimation, the disease is ranked fifth in terms of incidence and third in mortality, with regards to the total cancer cases worldwide. Currently, 1,033,700 new cases of GC are reported globally (equivalent to 5.7% of all cancer cases), out of which 783,000 (8.2%) die from the condition (Bray et al., 2018). At present, strategies being employed to treat GC includes surgery, chemotherapy, and molecular targeted therapy. But the therapeutic efficacy is not ideal and lead to a poor overall survival in GC patients. The use of conventional chemotherapy has not been very successful. Also, surgical resection has been associated with metastasis, as well as recurrence. Cancer stem cells (CSCs) have been implicated in poor treatment outcomes. CSCs, a subpopulation of tumors, take the main responsibility for the maintenance and spreading of tumor. Given that these cells have a high capacity to proliferate and self-renew, they generate many differentiated cells and normally are the main constituents of tumor population (Reya et al., 2001). Accumulating evidence suggests that gastric cancer stem cells (GCSCs) may play a crucial part in tumor recurrence, metastasis and therapeutic resistance (Xu et al., 2013; Stojnev et al., 2014). CSCs are resistant to traditional chemotherapy and radiotherapy, and can even form a larger proportion of the remaining GC cells at metastatic sites following chemotherapy (Brungs et al., 2016). As such, by targeting the key molecules that participates in CSC maintenance, we could eliminate CSCs and thus improve the prognosis of GC patients (Fu et al., 2020).

Stem cell features of cancer samples are quantitatively represented by mRNA expression-based stemness index (mRNAsi). By applying a one-class logistic regression machine learning algorithm (OCLR) to normal tissue-derived pluripotent stem cells and their differentiated progeny, the transcriptomic and epigenetic feature sets were extracted (Malta et al., 2018). Then, a multiplatform analysis of transcriptomes and methylomes was performed to identified stem cell signatures and quantify stemness. Finally, mRNAsi and the epigenetic regulation based-index (EREG-mRNAsi) were obtained and applied to the TCGA database (the stemness index workflow were described in https://bioinformaticsfmrp.github.io/PanCanStem_Web). Therefore, we obtained the stemness indices of each GC tissue.

Focus has mainly shifted to screening DEGs, and not exploring gene interactions. It is in studying how genes interact that we can reveal the correlations between genes with semblable patterns of expression. Weighted gene co-expression network analysis (WGCNA) is a systematic biology method comprehensively used to explore the connections between gene modules and cancers (Langfelder and Horvath, 2008; Chen et al., 2018; Tang et al., 2018). By constructing the WGCNA co-expression network, we observed that similarly expressed genes were in the same module. Then, we analyzed the link between each module and corresponding clinical phenotype, and finally determined the module with the most significant relation to clinical phenotype.

Based on the TCGA database and applying bioinformatic method, we identified key genes correlated with GC stemness by merging mRNAsi with WGCNA. After the least absolute shrinkage and selection operator (LASSO) Cox regression analysis, we chose nine genes for the construction of a risk model. The nine-gene risk model might be used as independent prognostic factors for predicting the disease outcomes of GC patients.

## Materials and Methods

### Data Collection and Study Design

**FIGURE 1:**
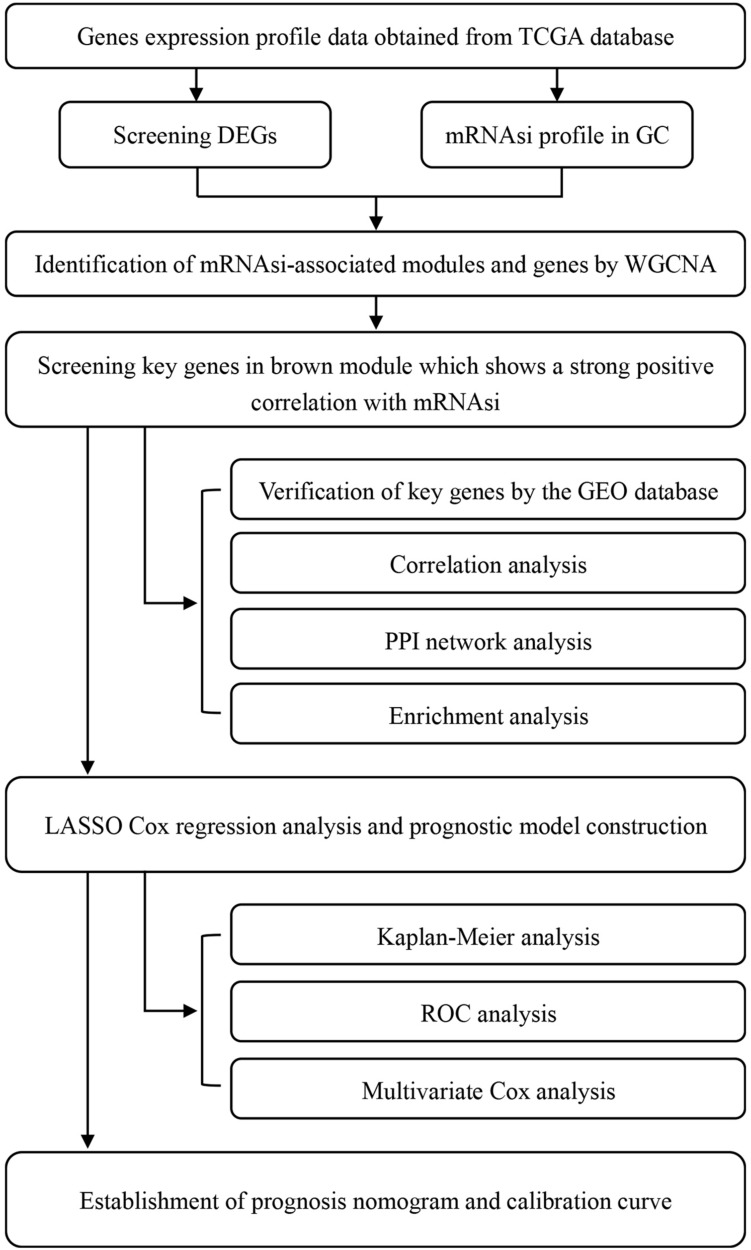
Flow chart of the study design. TCGA, The Cancer Genome Atlas; mRNAsi, mRNA expression-based stemness index; DEGs, differentially expressed genes; GC, gastric cancer; WGCNA, Weighted gene co-expression network analysis; GEO, Gene Expression Omnibus; PPI, protein-protein interaction; LASSO, least absolute shrinkage and selection operator; ROC, receiver operating characteristic.

We retrieved RNA-seq transcriptome data of GC cohort from TCGA database^1^ on February 28, 2020. These data comprised 375 and 32 samples of GC tissues and marching non-cancer tissues respectively. Also retrieved from the same database were the clinical data of 443 cases, which included gender, age, grade, TNM stages, pathological stage, survival time, and vital status. Subsequently, we merged the RNA-seq data of each sample into a matrix file with a merge script in the Perl language^2^. To change the names of the genes from Ensembl IDs to gene symbols, we utilized the Ensembl database^3^ in a matrix profile. The mRNAsi and EREG-mRNAsi indices of GC cases in TCGA were acquired from previous studies (Malta et al., 2018). The microarray (GSE29272, GSE27342, GSE26899) results for validation were downloaded from the Gene Expression Omnibus (GEO) database. As the data utilized herein were freely sourced from an open database, approval from the Ethics Committee was not required. As shown in Figure 1, our study design was briefly described in the flow chart.

### The mRNAsi Index Expression and Analysis of DEGs

The beeswarm package in R was employed to compare mRNAsi index in GC tissues versus non-cancer tissues. Similarly, DEGs in the two types of tissues were identified using the limma package (Ritchie et al., 2015). The selection criteria: | log_2_ fold change| > 1, *P* < 0.05 and false discovery rate (FDR) < 0.05. DEGs meeting the criterion were selected for further analysis. Heatmap and volcano plot were drawn using the pheatmap and limma packages, respectively.

### Weighted Gene Co-expression Network Analysis (WGCNA)

The WGCNA package was utilized to build a co-expression network targeting DEGs (Langfelder and Horvath, 2008). The dynamicTreeCut package, doParallel package, fastcluster package, foreach package, GO.db package, Hmisc packages, impute package, matrixStats package, preprocessCore package, and survival package were also used in WGCNA analysis.

We chose mRNAsi and epigenetically regulated mRNAsi (EREG-mRNAsi) as the representative traits to identify the CSC-associated modules and genes. Being a stemness index, mRNAsi, was generated from a group of stemness-associated epigenetically regulated genes. Modules related to the mRNAsi were selected, whose genes were considered to be co-expressed CSC-related genes. Initially, the normal data set and the cases with incomplete data were excluded (Supplementary Figure S1A). Subsequently, based on the gene expression levels of remaining samples, we clustered the data and reduced the outlier. A heatmap was generated to show the global outline of the mRNAsi and the EREG-mRNAsi expression in screened cases (Supplementary Figure S1B). Next, the power-value was chosen to construct a scale-free network based on the Pearson correlation coefficient among genes. The appropriate power-value = 4 was selected based on mean connectivity and scale-free correlation coefficient (Supplementary Figure S1C). Accordingly, we constructed a GeneTree and identified dynamic modules with a minimum size of 60 genes. On the GeneTree, branches of the cluster dendrogram corresponded to distinct gene modules and each piece of the leaves on the cluster dendrogram corresponded to a gene (Supplementary Figure S1D). Through further analysis of modules, the module eigengene (ME) dissimilarity was computed and visualized, then a cut-off (<0.25) was chosen for the module dendrogram and some similar modules were merged (Supplementary Figure S1E). Regarding principal component analysis (PCA), we considered MEs as the principal component of module for every gene. Particularly in some modules, each gene expression model was summarized to have distinct features.

To assess the significance of each module, we calculated the gene significance (GS) and analyzed the interaction between the levels of gene expression and sample characteristics. The calculation of GS was the log_10_ conversion of the *p*-value in the linear regression between gene expression and mRNAsi or EREG-mRNAsi (GS = lgp). In addition, the mean GS within the module was defined as Module significance (MS), which was determined to analyze the link between each module and sample characteristics. Among all selected modules, the module with the largest MS was taken as the module that is strongly related to sample characteristic.

Subsequently, GS and module membership (MM, relationship between genes in a given module and their expression profiles) for each gene and set their thresholds for screening key genes in the module as cor. gene GS > 0.5 and cor. gene MM > 0.8.

### Gene Correlation Analysis and Protein-Protein Interaction (PPI) Network Construction

The interactions between key genes at the level of transcription was analyzed by the Pearson correlation analysis with the corrplot package in R software. The PPI network of key genes was constructed using the online Search Tool for the Retrieval of Interacting Genes (STRING)^4^ (Szklarczyk et al., 2019). The bar-plot showed the nodes in the network with top connectivity. Based on this, we computed the sum of adjacent nodes of every gene in the PPI network. Next, using a bar plot, the genes were classified on the basis of adjacent node number. In addition, the pheatmap package was used to draw a heatmap showing the levels of expressions of key genes, and for plotting box-plots, the ggpubr package was utilized.

### Functional Annotation and Pathway Enrichment Analysis

The org.Hs.eg.db package was chosen to map the key genes with the Ensemble ID. The clusterProfiler package was performed to carry out GO functional annotation and KEGG analyses so as to explore and determine the potential biological functions of each key gene (Yu et al., 2012). The enrichplot package, colorspace package, stringi package, DOSE package and ggplot2 package were also used and the enriched biological processes (BP), cellular component (CC) and molecular function (MF) were obtained. Statistical significance was set at *P* < 0.05 and an FDR < 0.05. The bar-plot and the bubble-plot were drawn using R software in order to visualize the top results.

### LASSO Cox Regression Analysis and Construction of the Risk Assessment Model

The LASSO Cox regression analysis was conducted by the glmnet package and survival package to choose the most suitable genes for modeling. The LASSO regression is an approach for variable selection in fitting high-dimension generalized linear model. By constructing a penalty function with the LASSO regression, we could get a more refined model to decrease the variable numbers and successfully prevent overfitting. Herein, glmnet package was applied to determine the penalty parameter lambda via the cross-validation and identified the optimal lambda value which corresponded to the minimum value of the cross-validation error mean. Then, we chose the best gene group to construct a risk model and categorized the results into the high-risk or low-risk groups. We calculated the risk score based on a linear combination of the coefficients obtained from the LASSO Cox regression model multiplied with the expression value of each selected gene. The independent prognostic role of the risk model was analyzed using multivariate Cox regression. Finally, in order to offer a quantitative tool for predicting the individual probability of patient prognosis, we used the rms package to establish a prognosis nomogram and draw calibration curve to compare the expected and observed survival probabilities.

### Statistical Analyses

We completed the analyses using R software (R Core Team, 2013). Herein, all the cut-offs, comprising mRNAsi, expression levels of key genes, and risk score were the median level of each item. We applied the Wilcox test to assess the difference in mRNAsi scores between GC samples and normal samples. The difference in overall survival in low-versus-high risk score patients was analyzed using Kaplan-Meier analysis and log-rank test. The Kruskal-Wallis test was selected to examine correlation in risk scores versus clinicopathological characteristics. Univariate and multivariate analyses were conducted based on a Cox proportional hazard regression model. All data sets merging was performed with a merge script in the Perl language. Statistical significance was set at *P* < 0.05.

## Results

### The mRNAsi and DEGs Between GC Tissues and Non-tumor Tissues

**FIGURE 2:**
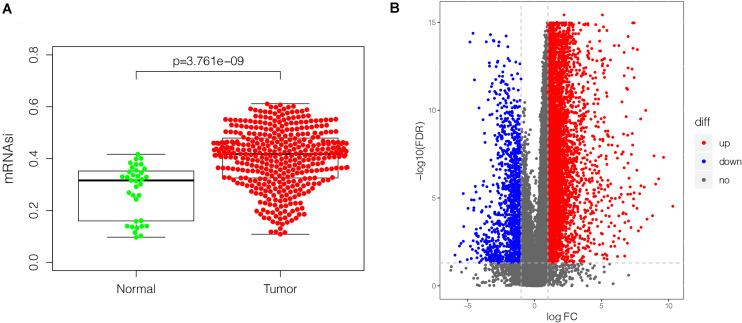
The mRNAsi and DEGs in GC (375 tumor tissues and 32 non-tumor tissues) based on TCGA database. **(A)** Differences in mRNAsi in GC tissues vs. non-tumor tissues. **(B)** volcano plot showing differential expression in GC tissues vs. non-tumor tissues. The upregulated gene is displayed in red dot and the downregulated gene is in blue. In total, 6,739 DEGs were identified, of which 5,593 were upregulated, and 1,146 were downregulated. mRNAsi, mRNA expression-based stemness index; DEGs, differentially expressed genes; GC, gastric cancer; TCGA, The Cancer Genome Atlas.

mRNAsi has been applied effectively to evaluate the tendency of tumor cells to dedifferentiate. As such it is used as a marker for identifying CSCs. A remarkably higher mRNAsi was recorded in GC tissues relative to non-cancer tissues (Figure 2A). The DEGs modulating tumor cell stemness were recognized after examining the RNA-seq data retrieved from the TCGA database. Out of the 6,739 DEGs screened, 5,593 were overexpressed, whereas 1,146 were under-expressed (Figure 2B, Supplementary Figure S2, and Supplementary Table S1).

### Discovering of the Most Significant mRNAsi-Related Modules and Genes

**FIGURE 3:**
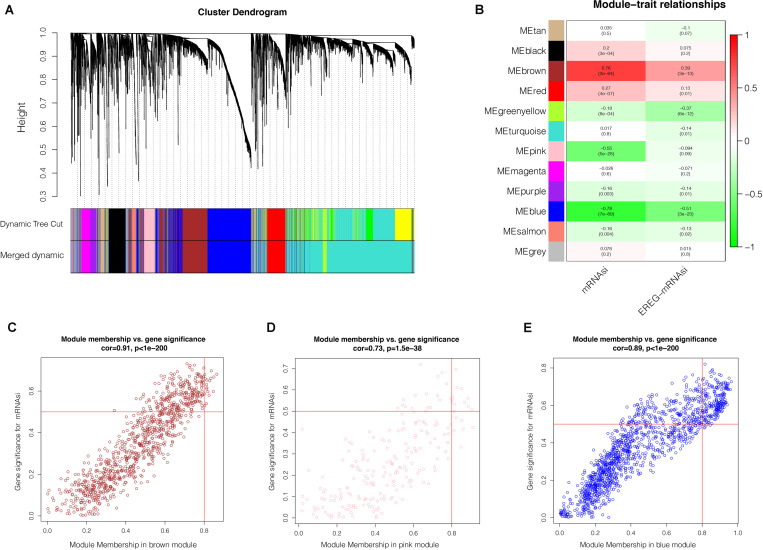
Weighted gene co-expression network analysis. **(A)** Co-expression module identification in GC. The branches of the cluster dendrogram represent the 12 different gene modules. Each module denotes a collection of co-related genes and was given a unique color. Each piece of the leaves on the cluster dendrogram represent a gene. **(B)** Heatmap displaying the correlations and significant differences between the gene modules and mRNAsi scores or EREG-mRNAsi. The upper row in each cell represent the correlation coefficient ranging from -1 to 1 of the correlation between a certain gene module and mRNAsi or EREG-mRNAsi. *P*-values are shown in brackets. Scatter plot of module eigengenes in the brown **(C)**, pink **(D)**, blue **(E)** modules. Each circle denotes a gene, and the circles in the upper right stand for the key genes in the modules. GC, gastric cancer; mRNAsi, mRNA expression-based stemness index.

**FIGURE 4:**
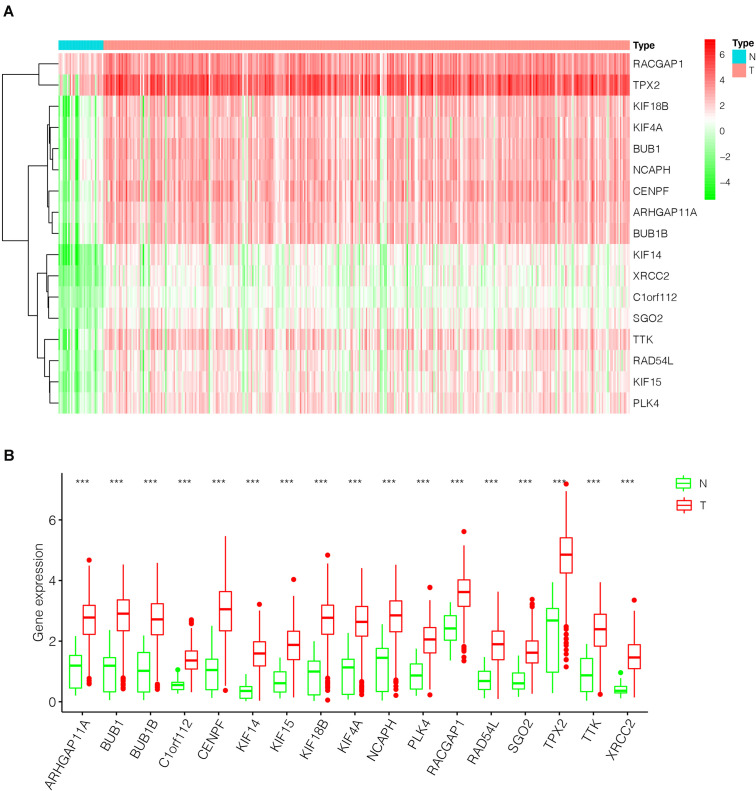
The differential expression of the key genes in GC (375 tumor tissues and 32 non-tumor tissues) based on TCGA database. **(A)** Heatmap of the key genes in the two groups. Red indicates upregulation, and green indicates downregulation. **(B)** Box-plot of the key genes in the two groups. ***P < 0.001; GC, gastric cancer.

**FIGURE 5:**
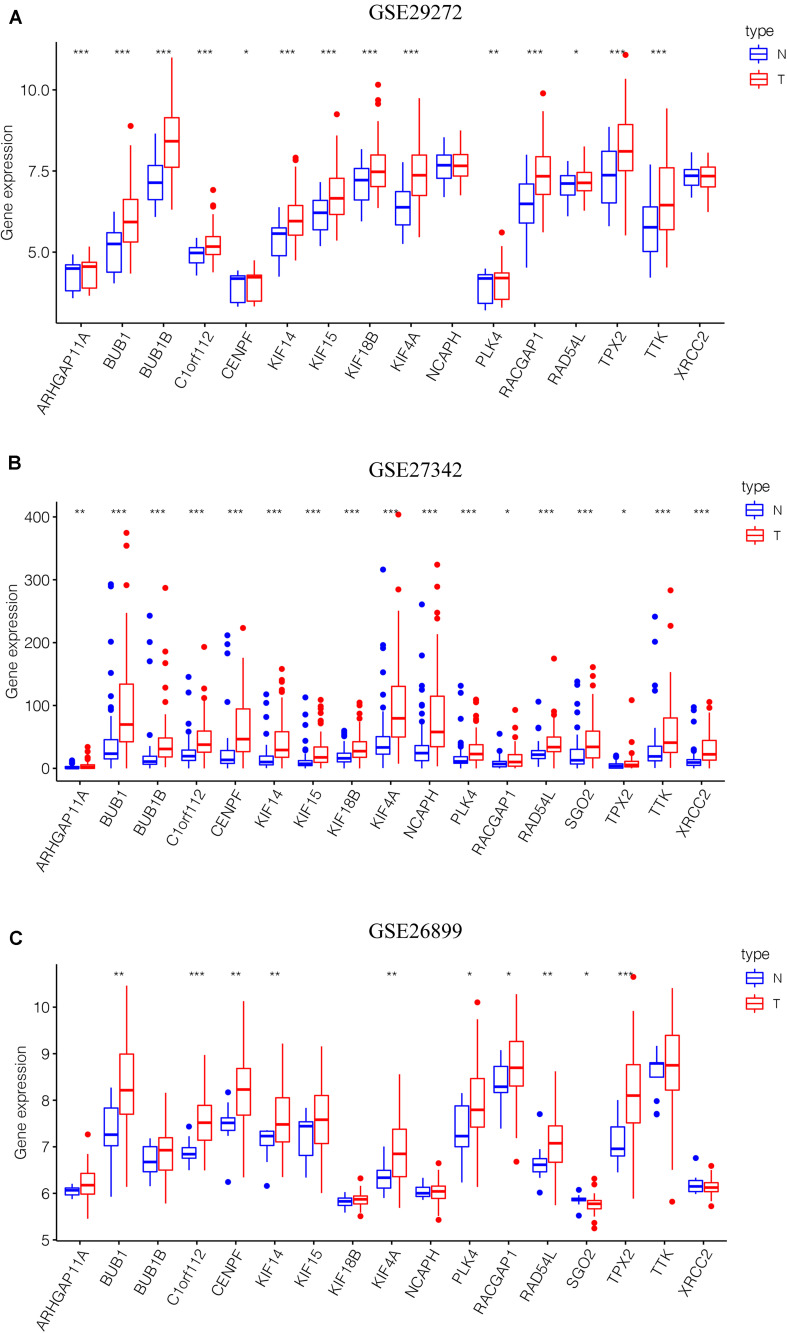
Validation of the key genes in the GEO microarray database. **(A)** GSE29272, 268 samples. **(B)** GSE27342, 160 samples. **(C)** GSE26899, 108 samples. *P < 0.05, **P < 0.01, *** *P* < 0.001. GEO, Gene Expression Omnibus.

After the screen of DEGs between GC tissues and non-tumor tissues, we constructed a gene co-expression network for the purpose of identifying the biologically significant gene modules by WGCNA, and to further identify genes strongly linked to GC stemness. In this study, a total of 12 modules were obtained for subsequent analysis (Figure 3A). Module significance (MS) was calculated to analyze the link between mRNAsi scores and gene. Due to that *R*^2^-value indicates the stronger the link between GC stemness and gene expression, the nearer the value was to 1. As shown in Figure 3B, three modules were considered the strong correlation to GC stemness, namely brown module, blue module and pink module. The brown module exhibited a positive correlation with mRNAsi (*R*^2^ = 0.76, *P* < 0.001) (Figure 3C), while the blue and pink modules reflected a negative correlation with mRNAsi (*R*^2^ = −0.78, *P* < 0.001; *R*^2^ = −0.55, *P* < 0.001, respectively) (Figures 3D,E). Thus, we chose the brow module for further analyses. The thresholds for selecting key genes in the module were defined as cor. gene MM > 0.8 and cor. gene GS > 0.5. Finally, we checked 17 key genes containing ARHGAP11A, BUB1, BUB1B, C1orf112, CENPF, KIF14, KIF15, KIF18B, KIF4A, NCAPH, PLK4, RACGAP1, RAD54L, SGO2, TPX2, TTK, and XRCC2. The concrete expression values of each key gene were extracted and the corresponding heatmap and boxplot were drawn, showing the upregulation of the key genes in GC tissues (Figures 4A,B). To verify the expression difference of key genes in the TCGA database, we chose the GEO dataset and selected three data sets (GSE26899, GSE27342, GSE29272) with large sample sizes to assess the key genes’ levels of expression in GC. Similar to the results obtained from the TCGA database, upregulation of all the key genes in the GC tissues was observed (Figures 5A–C).

### Correlation Analysis of Key Genes and PPI Network Analysis

**FIGURE 6:**
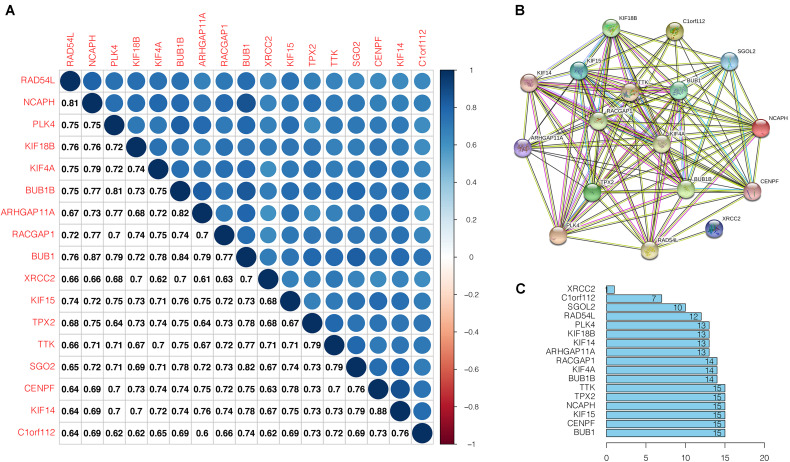
Correlation analysis of the key genes. **(A)** Correlation among the key genes at transcriptional level. Within the figure, the upper part shows the correlation strength based on the color, whereas, the lower part represents the matching correlation value. **(B)** Protein-protein interactions of the key genes. Circles denotes genes, lines indicate interactions between gene-encoded proteins, and line colors denote proof of associations between proteins. **(C)** Edge number of each key gene.

The correlation analyses of the key gene expression were carried out to confirm the relevance of the genes within the brown module. Within the figure, the strength of correlation is displayed on the upper part based on color, whereas the lower part represents the equivalent correlation value (Figure 6A). We found that strong positive correlation among the key genes at the transcription level (Pearson correlation ≥ 0.60). The relationship with the highest correlation was between KIF14 and CENPF (0.88), followed by BUB1 and NCAPH (0.87). BUB1 was highly correlated with NCAPH, BUB1B, and SGO2 (Pearson correlation > 0.80). At the protein level, the interactions between key gene proteins were analyzed using STRING and mapped the PPI network (Figure 6B). The PPI network consisted of 14 nodes and 214 edges, and 6 genes (TTK, TPX2, NCAPH, KIF15, CENPF, BUB1) have the highest node numbers (node number = 15). Except for XRCC2, other key genes showed a closer protein interaction (node number ≥ 7) (Figure 6C).

### Functional Annotation and Pathway Enrichment Analysis of Key Genes

**FIGURE 7:**
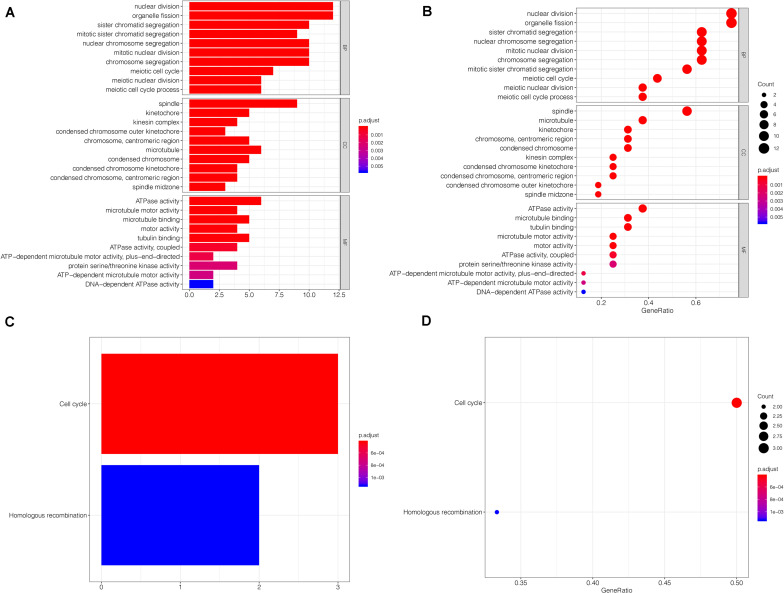
Functional annotation and pathway enrichment analysis. **(A,B)** The GO enrichment analysis of the key genes. Bubble-plots and Bar-plots showing the top terms in groups of BP, CC, and MF. **(C,D)** The top terms of the KEGG pathway analysis for the key genes are also shown by bar-plot and bubble-plot. Count: Number of genes linked to the enriched GO or KEGG pathway. BP, biological process; CC, cell component; MF, molecular function.

Regarding gene enrichment, the clusterProfiler package was employed to examine the functional link between the key genes. The GO enrichment analysis included the following three portions: biological process (BP), cell component (CC), and molecular function (MF) (Supplementary Table S2). The principal biological roles of the key genes were nuclear division (GO:0000280), organelle fission (GO:0048285), spindle (GO:0005819), and ATPase activity (GO:0016887) (Figures 7A,B). Regarding signaling pathway enrichment, the enriched pathways included cell cycle (hsa04110) and homologous recombination (hsa03440) (Figures 7C,D). The KEGG results are summarized in Supplementary Table S3.

### Construction of the Risk Assessment Model

**FIGURE 8:**
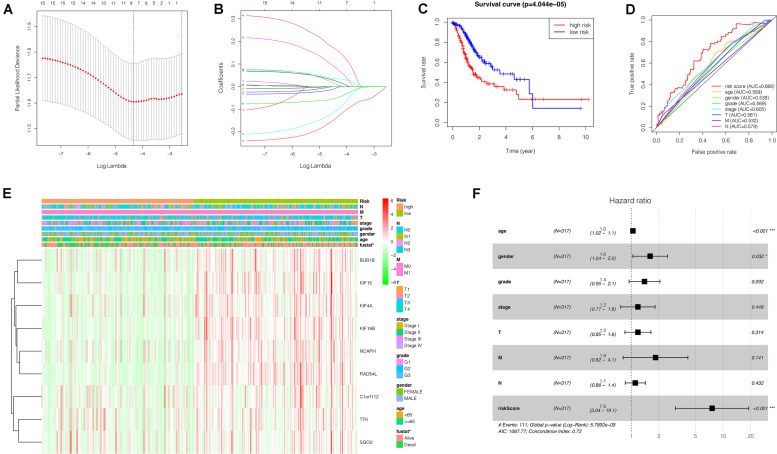
Construction of a nine-gene risk model and its prognostic value for GC patients based on TCGA database. **(A)** Selection of the optimal genes used to construct the final prediction model by LASSO regression analysis. Ten-fold cross-validation for tuning parameter selection. The number on top of the plot represents the total number of genes. Partial likelihood deviance is plotted against log lambda. Dotted vertical lines were drawn at the optimal values. The optimal gene group was chosen by 10-fold cross-validation and the minimal value of lambda. **(B)** LASSO coefficient profiles of the key genes. The number on top of the plot represents the total number of genes. Each curve represents corresponding key gene and the number next to it is the serial number of each gene. **(C)** Kaplan-Meier curve of the relationship between risk score and OS of GC patients. **(D)** The receiver operating characteristic (ROC) curve of the risk model for survival prediction. **(E)** The heatmap displays the expression of the nine genes and the correlation of clinicopathological parameters with different risk groups. Red indicates upregulation, and green indicates downregulation. **(F)** Forest plot for the multivariate Cox proportional hazard regression model of the risk score and clinicopathological parameters. GC, gastric cancer; OS, overall survival. *P < 0.05, ***P < 0.001.

To investigate the effect of key genes on GC prognosis, 17 key genes were input in LASSO regression to identify robust markers. Then, a prognostic model containing nine genes (BUB1B, NCAPH, KIF15, RAD54L, KIF18B, KIF4A, TTK, SGO2, C1orf112) constructed to evaluate the disease outcome of each patient (Figures 8A,B). We calculated the risk scores of GC patients with the LASSO Cox regression model according to the coefficients of nine genes.

Risk score = (BUB1B × 0.021265698)+(NCAPH × 0.00722087 6)+(KIF15 × −0.100943988)+(RAD54L × −0.133097331) + (KIF 18B × –0.048222701)+(KIF4A × −0.002827726)+(TTK × 0.03503 4586)+(SGO2 × 0.074640650)+(C1orf112 × 0.157469647).

### The Role of the Risk Model in GC Patient’s Prognosis

**FIGURE 9:**
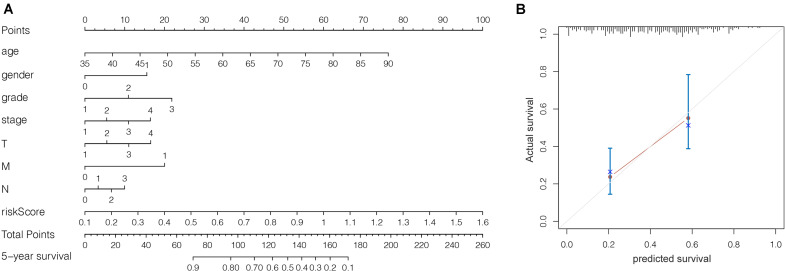
**(A)** Prognostic nomogram on the basis of risk score and clinical information. **(B)** The calibration curve of the prognostic nomogram. Dashed line at 45° represents perfect prediction.

We grouped the patients into two classes (low- and high-risk groups) based on the median risk score. The two groups exhibited substantial difference regarding the survival rate. Specifically, high-risk group had markedly lower 5-year survival rate (23.3%) in comparison to the low-risk group (43.1%) (*P* < 0.001) (Figure 8C). Notably, the low and high curves exhibited a remarkable intersection at the sixth year. In the first 6 years, the survival probability of high-risk patients was lower in comparison to low-risk patients and in the following 4 years, the survival probability curve was basically flat. The patients who exhibited high risk scores lived beyond 6 years, and, in exceptional cases, they even showed a higher probability of survival. The area under curve (AUC) of risk score was higher than that of clinicopathological characteristics (Figure 8D). The heatmap revealed substantial differences in high-risk versus the low-risk groups in fustat (*P* < 0.05) (Figure 8E). Also, the risk model performance in GC patients were further assessed by univariate and multivariate Cox regression analysis, which indicated that age, TNM stages, and risk score had a significant association with OS (*P* < 0.005) (Table 1). Multivariate analyses indicated that the nine-gene risk model is an independent predictor for predicting the disease outcomes GC patients (Table 1 and Figure 8F). A prognostic nomogram was established by incorporating data on risk score and clinical records to offer a quantitative tool that can be applied to predict the probability of relapse for each patient (Figure 9A). Age, gender, grade, TNM stages, and risk score were parameters included in the nomogram. The calibration curve of the prognostic nomogram showed good agreement between prediction and observation (Figure 9B).

**TABLE 1:** Univariate and multivariate Cox regression analyses of the association between clinicopathological parameters and overall survival in GC patients.

Parameter	Univariate analysis			Multivariate analysis		
	HR	95%CI	*P*	HR	95%CI	*P*
Age	1.027	1.008–1.046	**0.006**	1.043	1.022–1.064	**0.000**
Gender	1.484	0.980–2.247	0.062	1.606	1.042–2.476	**0.032**
Grade	1.368	0.947–1.977	0.095	1.394	0.947–2.054	0.092
Pathological stage	1.535	1.221–1.931	**0.000**	1.182	0.768–1.818	0.448
T	1.298	1.023–1.645	**0.032**	1.183	0.853–1.640	0.314
N	1.267	1.069–1.502	**0.006**	1.107	0.860–1.424	0.432
M	2.048	1.096–3.827	**0.025**	1.839	0.816–4.143	0.141
Risk score	7.226	2.949–17.707	**0.000**	7.606	3.037–19.051	**0.000**

*Bold values indicate P < 0.05. HR, hazard ratio; CI, confidence interval; GC, gastric cancer.*

## Discussion

Although the classical cancer therapies, including chemotherapy and radiation therapy, have contributed to the improvement of cancer treatments. However, for most cancer patient’s disease recurrence is a common event. To achieve a better therapeutic effect, the research of CSCs is springing up vigorously recent years. Obtained findings showed that CSCs, similar to other stem cells being capable of self-renewal and multipotent differentiation (Han and Oh, 2013), are considered as the reason behind the continuous proliferation, as well as recurrence of cancers. With the potential to initiate and sustain tumor progression, CSCs are involved in tumor progression, metastasis and therapeutic resistance in GC (Kreso and Dick, 2014; Brungs et al., 2016; Chang, 2016; Çoban and Şahin, 2018). However, no relevant therapeutic modalities targeting CSCs have been developed yet. Therefore, it is reasonably urgent and important to recognize the key genes that can be used as the therapeutic targeting of GCSCs. Herein, our focus was on the key genes related to GCSCs using WGCNA based on an mRNAsi index, as calculated by Tathiane et al. via the OCLR algorithm. Firstly, we analyzed the difference in mRNAsi scores in GC tissues versus non-tumor tissues. GC tissues exhibited a higher stemness in comparison to non-tumor tissues, and this was in accordance with other recently published reports in bladder cancer stem cell (Pan et al., 2019), lung adenocarcinoma stem cell (Zhang et al., 2020), and breast cancer stem cell (Pei et al., 2020).

WGCNA is a tool to analyze the complex correlations between genes and phenotypes by transforming gene expression data into co-expression module. By virtue of WGCNA, we can group the genes based on their patterns of expression and then analyze the link between various gene clusters and clinical phenotypes (Langfelder and Horvath, 2008). In this study, we used WGCNA to make weighted connection analysis of DEG expression profiles in GC tissues and non-tumor tissues, and preliminarily divided DEG into different gene clusters. Therefore, the genes which were highly co-expressed produced a gene module that might be applied to assess the depth of the correlation between the gene modules and selected clinical characteristics. We selected the brown modules with the strongest correlations with mRNAsi, where 17 Key genes were vetted on the basis of the GS and MM scores. Being upregulated in GC tissues, all these key genes displayed not only a strong association between their proteins but also a strong association with regards to co-expression at the level of transcription. This suggests that the key genes may have strong biological links and are synergistic in their functions. We conducted GO functional and KEGG pathway enrichment analyses for key genes. According to functional enrichment, the genes were clustered mostly in the functional set associated with cell mitosis, suggesting that these genes could have a role in enhancing the self-renewal and proliferative characteristics of stem cells. As such, by targeting these genes, we could suppress these characteristics of GCSCs. Regarding pathway enrichment, cell cycle pathway was the main point of concentration, indicating that the key genes could influence the tumor stemness through cell cycle modulation. Many studies have implicated these key genes in GC pathogenesis, with suggestions that they may be directly linked to the CSCs features. Cell cycle is a tightly regulated process involving the participation of several genes, for example, a spindle damage during the cell cycle progression leads to abortion of the process as mediated by BUB1, which function at spindle assembly checkpoint to suppress cell cycle progression (Shigeishi et al., 2001). As such, BUBI downregulation is an independent prognostic marker in GC (Stahl et al., 2017). However, BUB1 is not the specific biomarker for GC. In fact, it is also closely associated with tumorigenic phenomena in many other cancers. As Piao et al. (2019) demonstrated, BUB1 may play a role in the progression of pancreatic ductal adenocarcinoma and could serve as a prognostic biomarker for patients with pancreatic ductal adenocarcinoma. Han et al. (2015) linked BUB1 to the features breast cancer stem cells. BUB1B and TTK may contribute to gastric tumorigenesis and risk of tumor development (Hudler et al., 2016). Yet BUB1B is also correlated with the progression and prognosis in patients with other cancers (Fu et al., 2016; Yang et al., 2015), and has been linked to CSC progression, radiation resistance (Ma et al., 2017). TTK gene is upregulated in a population of CSC-like cells extracted from human esophageal carcinoma. The same phenomenon occurs in the stem cells of human multiple myeloma (Huang et al., 2009; Zhou et al., 2014). CENPF is associated with GC proliferation and tumor metastasis (Chen et al., 2019) and may be an important regulator of prostate cancer (Shahid et al., 2018). Yang et al. linked the upregulation of KIF14 in GC to poor prognosis, suggesting that it could play a critical function in GC pathogenesis (Yang et al., 2019). In addition, KIF14 has been reported to serve oncogenic roles in a variety of malignancies such as colorectal cancer (Wang et al., 2018), medulloblastoma (Li et al., 2017), cervical cancer (Wang et al., 2016). Moreover, KIF15 played a critical role in inhibiting GC cell apoptosis and promoting cell proliferation, and the high expression of KIF15 predicts a poor prognosis in GC patients (Ding et al., 2020). Biljana et al. demonstrated that KIF15 upregulation in stem cells of glioblastoma is related to poor disease outcomes (Stangeland et al., 2015). PLK4 expression was upregulation in human primary gastric cancer, and it associated with the regulation of centrosome and stability of chromosome in GC (Shinmura et al., 2014). Additionally, PLK4 could be a therapeutic target for colorectal cancer (Liao et al., 2019) and a potential prognostic factor in breast cancer (Li et al., 2016). RACGAP1 is a modulator of the canonical Wnt signaling pathway, which participates in the pathogenesis of CG (Bornschein et al., 2016). Upregulation of RACGAP1, especially at the invasive front in GC has a strong link to factors that are related to cancer progression, as well as poor disease outcomes (Saigusa et al., 2015). TPX2 expression has been linked to cancer progression and poor survival in GC (Tomii et al., 2017), and closely related to the proliferation of breast cancer stem cells (Huang et al., 2017). In summary, these genes have been shown to correlate with the biological behaviors and poor prognosis of numerous cancers including GC, but they have been previously ignored in GCSCs, and may be targeted to suppress GC stemness features. However, given that these findings are based on bioinformatics involving retrospective data, there is a need to conduct further studies to confirm our conclusions.

Finally, we used the key genes to predict the disease outcomes of GC patients. LASSO is commonly applied algorithm which allows one to select and shrink variables simultaneously, thus enabling the identification of prognostic signatures (Bøvelstad et al., 2007). Simultaneous variable estimation as well as selection is achieved in LASSO through selecting compression coefficient absolute value then adjusting the lambda parameter. In LASSO, we can remove unnecessary variable coefficients by incorporating a constraint condition to the coefficients’ absolute value. Through that, we can establish a more refined model (Geng et al., 2020). After LASSO analysis, nine dysregulated and prognostic genes were identified. We constructed a nine-gene risk model that put GC patients into low-risk and high-risk groups. The high-risk group exhibited poorer overall survival. Univariate and multivariate Cox analysis verified that the nine-gene risk model is an independent predictor for predicting the disease outcomes of GC patients.

## Conclusion

In conclusion, we have discovered 17 key genes related to GCSCs using WGCNA based on an mRNAsi index. These genes were of pivotal importance in GC stem cell maintenance and could be potential therapeutic targets for inhibiting GC stemness characteristics in clinical application. Furthermore, we constructed a nine-gene-based prognostic model by LASSO regression, which can be applied to predict the disease outcomes of patients. The prognostic nomogram combined with nine-gene model and clinicopathological parameters could be expected to serve as an accurate and efficient tool to assess the prognosis of GC patients for clinicians, which might be beneficial for individualized treatment and medical decision making. Based on our literature such, we believe that the study herein is the first to report a novel GCSC biomarker (mRNAsi), which can be used to determine GC progression. However, conclusions were derived from bioinformatic analysis of retrospective data, and therefore, is a need to conduct further studies to confirm our conclusions.

## Data Availability Statement

All datasets presented in this study are included in the article/Supplementary Material.

## Author Contributions

XC conceptualized and designed the study, analyzed and presented the data, and drafted the manuscript. DZ collected data and did some statistics. FJ and YS retrieved the data from various databases. XL and XH participated in data analysis. PW and XS led the study and read the first draft of the manuscript. The final draft was verified by all authors before the submission.

## Conflict of Interest

The authors declare that the research was conducted in the absence of any commercial or financial relationships that could be construed as a potential conflict of interest.

## Footnotes

1 https://portal.gdc.cancer.gov

2 http://www.perl.org/

3 http://asia.ensembl.org/index.html

4 https://string-db.org

## Supplementary Material

FIGURE S1 **(A)** The data over the red line were the normal data and the missing data, and they were deleted for the outlier elimination. **(B)** The heatmap shows the global overview of the mRNAsi values and the EREG-mRNAsi values. **(C)** Considering the scale-free correlation coefficient and mean connectivity, the appropriate power value = 4 was selected. **(D)** A GeneTree was constructed according to the power value. The branches of the cluster dendrogram correspond to the 12 different gene modules. Each piece of the leaves on the cluster dendrogram represent a gene. **(E)** Calculating the module similarity to join the modules with high similarity.

FIGURE S2 Heatmap of differentially expressed genes. Red indicates upregulation, and green indicates downregulation.
