# Supplementary figures and images for: Prognostic Prediction Using a Stemness Index-Related Signature in a Cohort of Gastric Cancer
Source: Front Mol Biosci. 2020 Sep 4;7:570702. doi: 10.3389/fmolb.2020.570702 (PMC7504590; doi:10.3389/fmolb.2020.570702)

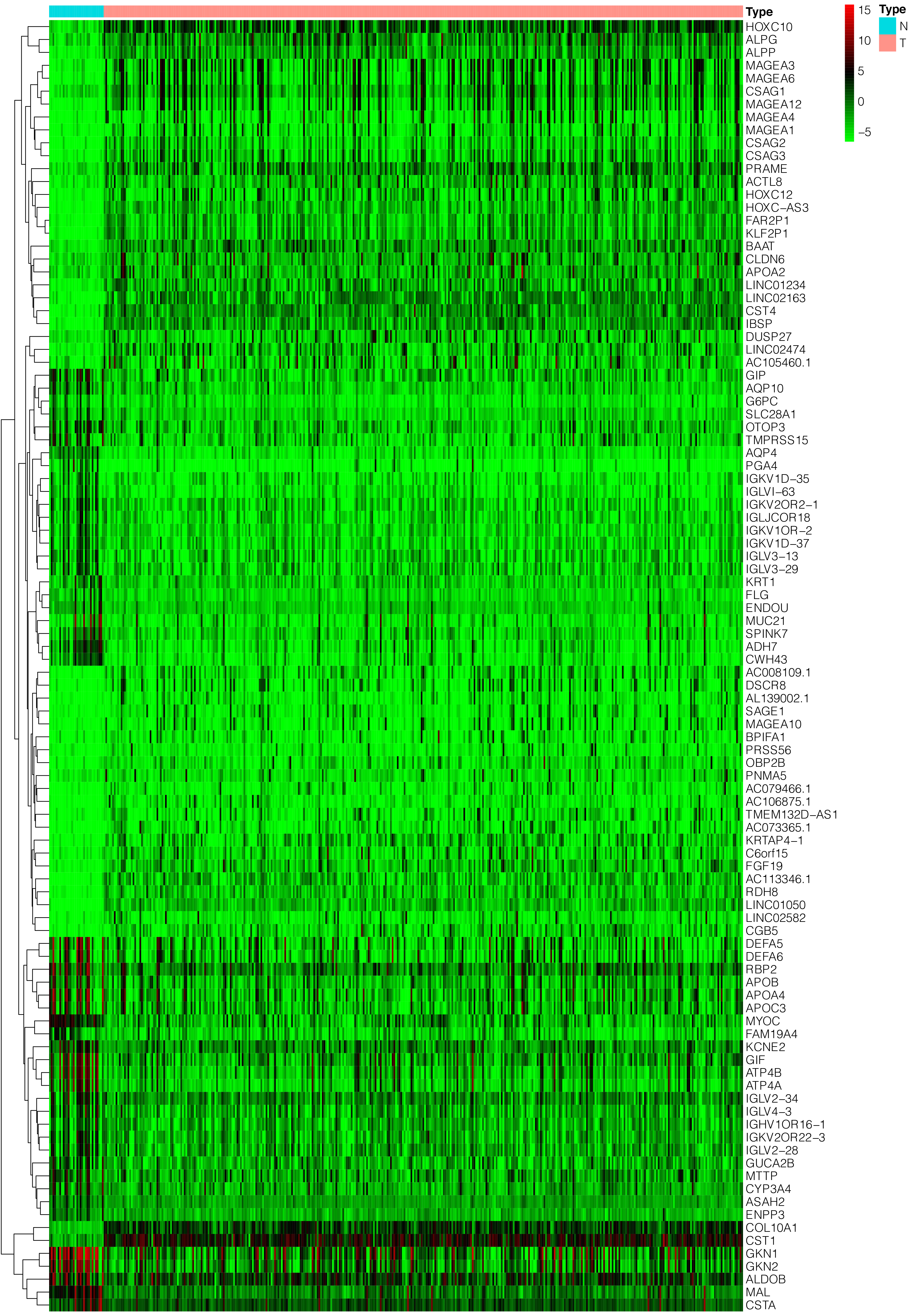

Supplement: FIGURE S2 — Heatmap of differentially expressed genes. Red indicates upregulation, and green indicates downregulation. [file Image_2.JPEG]
